# Intrinsic elaboration of prefrontal modularity: a dual-control model of axon bundling and synaptic docking

**DOI:** 10.3389/fnana.2026.1761080

**Published:** 2026-06-02

**Authors:** Tetsuo Yamamori, Akiya Watakabe, Henrik Skibbe

**Affiliations:** 1 Department of Biological Function and Regulation, CIEA (Central Institute for Experimental Medicine and Life Science), Kawasaki, Japan; 2 Laboratory of Haptic Perception and Cognitive Physiology, Center for Brain Science, RIKEN, Wako, Japan; 3 Laboratory for Molecular Mechanisms of Brain Development, Center for Brain Science, RIKEN, Wako, Japan; 4 Faculty of Informatics, Department of Informatics, Matsuyama University, Matsuyama, Japan; 5 Brain Image Analysis Unit, Center for Brain Science, RIKEN, Wako, Japan

**Keywords:** axon and dendrite development, axon bundles, axon guidance cue, gradient, prefrontal cortex, retinoic acid, synaptogeneisis, cortical areas

## Abstract

The evolutionary expansion of the primate prefrontal cortex (PFC) presents a profound biological enigma: how does this region achieve a highly ordered, modular architecture in the absence of direct dense sensory templates that govern primary sensory areas? In this review, we synthesize classical neuroanatomical frameworks with recent advances in spatial transcriptomics and connectomics to delineate a model of intrinsic elaboration. We propose that PFC modularity emerges from a developmental program facilitated by expansion of the outer subventricular zone (OSVZ) and the legacy of whole-genome duplication (2R-WGD). Central to this proposal is a “Dual-Control Model” of circuit assembly, inferred by integrating anatomical tracer data with spatial and spatiotemporal transcriptomic datasets. This framework suggests that long-range connectivity is established through pre-target axon bundling (fasciculation), governed by a high-dimensional navigation code (e.g., ephrin/Eph, PCDH11X, PCDH17, ROBO2), while these bundles are anchored onto vertical columnar scaffolds through synaptic docking mechanisms (e.g., CBLN2, cadherins). By contrasting the PFC with the map-driven visual system, point-driven olfactory system, and layer-driven hippocampus, we argue that PFC uniqueness lies not in novel genes but in a combinatorial logic of a shared molecular toolkit, which can be understood as intrinsic elaboration. This framework may facilitate the emergence of a cognitive scaffold under relatively weak external sensory constraints. These molecular systems are considered to operate in concert with activity-dependent developmental refinement rather than independently of neural activity.

## 1 Introduction

The evolutionary expansion of the primate prefrontal cortex (PFC) presents a profound biological enigma: how does this region achieve a highly ordered, modular architecture in the absence of the dense sensory templates that govern primary sensory areas? In the primary visual cortex (V1), columnar organization is increasingly understood as a “passive geometric emergence” stabilized by high-density retinal inputs and competitive synaptic pruning (Jang et al., 2020; Rakic, 1988). In stark contrast, the PFC lacks such extrinsic templates, yet it develops a sophisticated columnar lattice essential for high-dimensional cognition. In this review, we synthesize classical neuroanatomical pillars with recent breakthroughs to delineate a model of intrinsic elaboration. We argue that the PFC does not simply “copy” the sensory-driven logic of the visual system (Drescher et al., 1995; Cheng et al., 1995; Cang et al., 2005; Ahmed et al., 2011), but instead employs a developmental program with stronger intrinsic molecular contributions. By examining the synergy between genetic constraints, specialized proliferative zones, and a unique Dual-Control Model of axon bundling and synaptic docking, we highlight the PFC as one of the brain’s most highly organized association architectures, a region in which intrinsic molecular and developmental programs may contribute more strongly to cortical organization under relatively weak external sensory constraints.

## 2 Section 1: determinants and constraints of PFC modularity

### 2.1 Genetic constraints and the intrinsic program

The evolutionary development of the primate prefrontal cortex (PFC) occurs despite a high degree of stability in the protein-coding genome across mammalian lineages. While mammalian orders exhibit significant diversity, the number of structural, protein-coding genes remains consistent at approximately 26,000. This suggests that major macroevolutionary shifts in neural architecture are driven not by new coding genes, but by genomic innovations such as the legacy of two-round whole-genome duplication (2R-WGD; Dehal and Boore, 2005) and the expansion of the non-coding regulatory landscape. We propose that the PFC achieves stable modularity through intrinsic elaboration, a genetically constrained program where intrinsic molecular mechanisms stabilize internal cognitive scaffolds. This process prevents the stochastic “salt-and-pepper” organization typically found in the absence of high-density sensory inputs (see Table 1).

**Table 1:** Proposed molecular candidates for the PFC dual-control model.

System	Candidate molecules	Proposed role	Evidence level	Basis for assignment
Wiring-related systems	PCDH11X/17, ROBO2, Ephs/ephrins	Axon fasciculation,topographic targeting, spatial projection organization	Inferred from convergent datasets	Spatial transcriptomics + known axon guidance functions
Plug-in-related systems	CBLN2, cadherins (e.g., CDH 8), SYT10	Synaptic stabilization, dendritic integration, local circuit refinement	Supported/inferred	Developmental and synaptic-organizer studies
Integration across scales	Interaction of wiring and plug-in systems	Integration of molecular signaling, synaptic organization, and large-scale connectivity	Hypothesis-generating framework	Present integrative model

This table presents an illustrative example based on the 8Ad-related association areas pathway to demonstrate how candidate molecular systems may be conceptually assigned within the proposed dual-control framework. Assignments are derived from the integration of anatomical connectivity, developmental studies, and spatial transcriptomic datasets. The table is intended to illustrate the logic of candidate molecular assignment rather than to provide definitive gene-level validation. Evidence levels are indicated to distinguish experimentally supported findings from hypothesis-guided inferences. Candidates labeled as “inferred” are based on convergent transcriptomic enrichment patterns and established functional properties of molecular families involved in axon guidance, synaptic organization, or dendritic stabilization. This framework should therefore be regarded as a hypothesis-generating model linking molecular signaling to circuit organization and cortical modularity.

### 2.2 OSVZ expansion and the genesis of Micro-columns

We propose that prefrontal organization is established through a process of intrinsic elaboration, wherein endogenous molecular signaling and structural constraints refine cortical architecture through intrinsic developmental mechanisms. This represents a potential transition from systems governed by passive geometric necessity to those guided by a coordinated group of molecular organizers. Recent research offers additional insights into the molecular, connectomic, and functional underpinnings of this refinement process.

### 2.3 Geometric constraints and the transition to columnarity

The structural divergence between the disordered salt-and-pepper organization in rodents and the columnar maps observed in primates is determined by phylogenetic shifts in geometric constraints (Horton and Adams, 2005). This process is defined as a geometric transition or a phase transition toward geometric order. The sampling model proposed by Jang et al. (2020) suggests a structural continuum between disordered and columnar organizations. This progression spans from the disordered rodent V1 to the clustered auditory and somatosensory cortices in rodents (Kanold et al., 2014), as well as the emerging columns observed in the rodent retrosplenial cortex and PFC. In contrast to V1, where organization is stabilized by high-density thalamocortical inputs, the PFC lacks such dense external templates. Instead, its organization (Wyss et al., 1990; Watakabe et al., 2023) is thought to emerge through intrinsic molecular and developmental mechanisms together with activity-dependent refinement. The primate PFC occupies a position along this trajectory where molecular programs refine these templates into stable cognitive macrocolumns. This continuum suggests that the primate PFC structure evolves from passive geometric mapping toward a cognitively specialized cortical architecture.

### 2.4 Universal structural solutions

Geometric logic is supported by the identification of orientation pinwheels in visual marsupials such as the wallaby (Jung et al., 2022). The existence of these structures in a lineage that diverged from placental mammals over 160 million years ago suggests that columnar organization is a universal structural solution to the problem of high-dimensional spatial sampling. This transition is not solely a function of brain size but involves a functional trade-off between local connectivity and global mapping efficiency. As cortical surface area and input density increase, columnar arrangements provide an energy-efficient solution for minimizing wiring length while maintaining continuous sensory representations (Jang et al., 2020). In primates, the thickness of layers 2 and 3, which results from the expansion of the outer subventricular zone (OSVZ), is significantly greater than in rodents (Smart et al., 2002; Kaschube, 2014). Sensory-input-dependent columnar properties are defined here as a form of self-organization that emerges through direct dependence on extrinsic sensory input. Such geometric transitions may provide a general architectural framework for columnar organization in sensory cortices. In contrast, the primate PFC appears to operate under relatively weaker external sensory constraints, where intrinsic molecular programs may contribute more strongly to the refinement and stabilization of large-scale association architectures.

## 3 Section 2: the foundational logic of columnar modularity and intrinsic refinement

The structural specialization of the primate PFC is not merely a product of increased volume, but is anchored in a rigorous modular architecture that defines the neocortex’s computational essence. Long before the molecular era, the “module-concept” proposed by Szentágothai (1975) established that the cortex is organized into discrete, vertically oriented units, repeating lattices of cells that serve as the basic computational building blocks. This was synthesized into the “Columnar Hypothesis” by Mountcastle (1997), who argued that this modularity is the universal principle of neocortical organization. While the functional architecture of the visual cortex, characterized by the ocular dominance columns described by Hubel et al. (1977), provided a “passive template” driven by sensory input, the primate PFC evolved to utilize this modular scaffold for cognitive processing.

The developmental origin of this modularity is rooted in the “Radial Unit Hypothesis” formulated by Rakic (1988), who proposed that the columnar organization of the adult cortex reflects the spatial arrangement of progenitor cells in the embryonic proliferative zones. In the primate PFC, the massive expansion of the outer subventricular zone (OSVZ), discussed in Section 1, directly increases the number of these radial units, thereby providing an expanded “horizontal lattice” of columns. This developmental hardware allows for a vast multiplication of the modular units available for higher-order integration.

The transition from a general cortical module to a PFC-specific modularity was pioneered by the Patricia Goldman-Rakic’s group (Goldman-Rakic and Schwartz, 1982; Goldman-Rakic, 1987). They demonstrated that the PFC is organized into rigorous modular clusters, where neurons with shared functional properties (such as spatial working memory) are grouped into vertical columns. Critically, these columns are not isolated; as shown by Kritzer and Goldman-Rakic (1995), the PFC possesses an intrinsic, periodic (stripe-like) circuitry characterized by horizontal clusters of axonal terminals. These “stripes” represent a highly organized system of long-range intrinsic connections that link distant modules, providing the anatomical foundation for the high-dimensional “fields” of interaction seen in the primate association cortex.

This macro-scale modularity is further empowered by the extraordinary complexity of its individual cellular components. Quantitative studies by Elston (2000, 2003) have established that pyramidal cells in the primate PFC possess significantly larger and more branched basal dendritic arbors, with spine densities nearly an order of magnitude higher than those in V1 (Elston and Rockland, 2002; Elston et al., 2011). This “cellular richness” is complemented by the electron microscopic analyses of DeFelipe (2011), which highlight the unique synaptic organization and increased density of excitatory synapses in human and non-human primate association cortices. These findings suggest that PFC columns provide an expanded synaptic integration capacity for integrating information within the modular scaffolds established earlier in development.

Finally, the maturation of these complex circuits follows a structural hierarchy. Barbas and Pandya (1989) established that PFC connectivity follows a logic where the degree of granularity and laminar organization determines the patterns of long-range projections. The selective stabilization of these hierarchical circuits involves a protracted period of developmental refinement. As emphasized by Innocenti (1995, 2011), the pruning of exuberant synapses and the elaboration of long-range bundles are essential for the emergence of functional modularity. In the primate PFC, this developmental window is significantly extended, a phenomenon that allows for the intrinsic refinement of columns under relatively weak external sensory constraints. Together, these classical pillars, from Rakic’s radial units and Goldman-Rakic’s periodic stripes to Elston’s synaptic complexity, provide the necessary structural substrate for the intrinsic elaboration of prefrontal circuitry (Supplementary Table 1).

## 4 Section 3: molecular and functional mechanisms of intrinsic refinement

**Figure 1:**
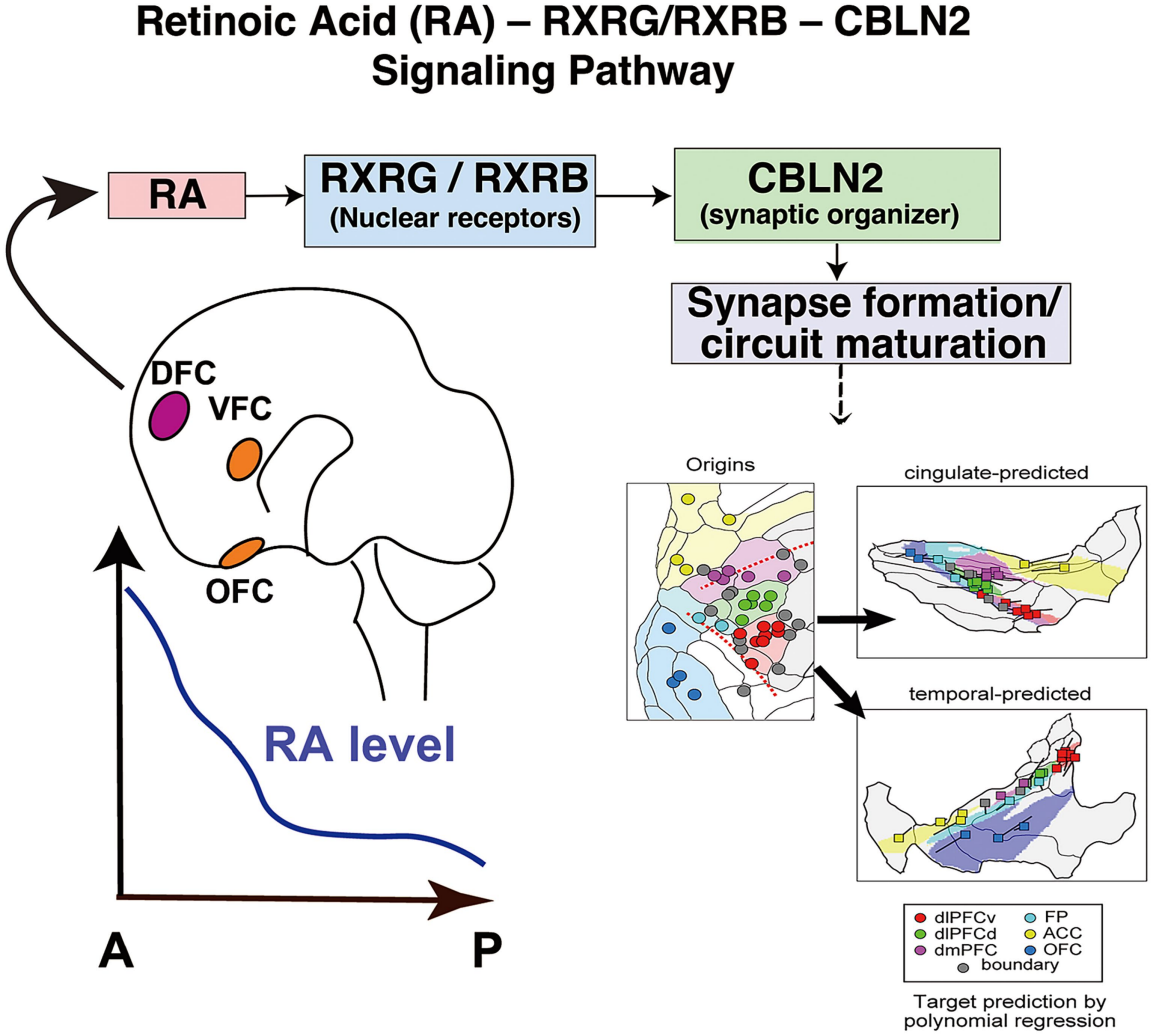
Integration of molecular signaling and structural hierarchy in cortical column formation. Left: Molecular programs underlying spinogenesis in the primate prefrontal cortex (PFC). Retinoic acid (RA) activates nuclear receptors RXRG/RXRB, up-regulating CBLN2, a synaptic organizer that promotes spinogenesis in PFC neurons. This RA–RXR–CBLN2 pathway, enhanced in humans (Shibata et al., 2021a, 2021b), supports the exceptionally high spine density of layer III pyramidal cells. Center: Synaptic proliferation and dendritic integration link molecular signaling to microstructural organization. Right (bottom panels): The inset on the left shows the injection sites, which are color-coded according to six distinct subregions (indicated by the box at the bottom right). The panel on the right shows columnar projections to the cingulate gyrus (top) and the temporal regions, which are represented by high-intensity signals. Polynomial regression analysis revealed distinct projection patterns between the two regions. Adapted from Watakabe et al. (2023), illustrating both columnar and diffuse projection patterns. This suggests that related developmental principles may contribute to distinct classes of projection systems to the same region.

The transition from a structural scaffold to a functional columnar map is guided by specific molecular organizers that facilitate endogenous refinement. A pivotal regulator in this process is the Retinoic Acid (RA)-CBLN2 signaling pathway (Figure 1). As demonstrated by Shibata et al. (2021b), the up-regulation of CBLN2 by RA through nuclear receptors (RXRG/RXRB) promotes spinogenesis and stabilizes excitatory synapses within the primate PFC. This molecular signature, potentially driven by primate-specific enhancers, provides the necessary cues for synaptic docking and stability, linking genetic constraints to large-scale architectural patterns.

Recent advances in spatial transcriptomics and connectomics have offered further insights into how these molecular programs manifest as organized modules (Supplementary Tables 1, 2). High-resolution spatial transcriptomics has identified discrete, periodic gene-expression “stripes” in the developing macaque PFC, characterized by the enriched expression of synaptic-associated molecules such as CBLN2 and SYT10 (Shibata et al., 2021b; Zhang et al., 2026). These molecular patches emerge prior to extensive associative input integration and align with the macro-columnar dimensions (approx. 0.5 mm) identified by classical studies. Furthermore, advanced connectomic tracing using viral-mediated barcoding has revealed structured and selective patterns of neuronal connectivity at single-cell resolution (Yuan et al., 2024), extending the concept of “nested modularity” (Watakabe et al., 2023). Supported by the extended developmental window of neoteny (Libé-Philippot et al., 2024), these findings suggest that the PFC possesses an inherent capacity to support the intrinsic refinement of prefrontal circuitry.

## 5 Section 4: a dual-control model linking connectivity architecture to molecular programs

Recent advances in large-scale tracer mapping and spatial transcriptomics provide an opportunity to bridge anatomical connectivity with molecular regulation in the primate prefrontal cortex (PFC). These organizational principles are broadly consistent with classical corticocortical pathway analyses in primates (Schmahmann and Pandya, 2006). High-resolution mapping studies in the marmoset have revealed that corticocortical projections consist of two complementary patterns: spatially restricted, columnar “patchy” projections and broadly distributed “diffuse” projections (Watakabe et al., 2023). These projection types are organized both locally and globally, reflecting underlying topographic gradients across the PFC.

Building upon this anatomical framework, recent transcriptomic and epigenomic studies have identified molecular gradients and developmental programs that parallel these structural hierarchies. For example, primate PFC development is characterized by prolonged synaptogenesis, region-specific gene expression, and coordinated neuron–glia interactions (Chen et al., 2023; Zhang et al., 2026). In particular, molecular systems such as the retinoic acid (RA)-CBLN2 pathway regulate dendritic spine formation and synaptic organization in a temporally and spatially controlled manner (Shibata et al., 2021a, 2021b; see Figure 1 for a schematic overview).

### 5.1 Conceptual framework: dual modes of circuit organization

Based on the integration of these anatomical and molecular findings, we propose a dual-control model of PFC organization, consisting of two complementary systems:

(i) “Wiring” systems: molecules that guide axonal targeting and establish spatially structured, columnar connectivity.
(ii) “Plug-in” systems: molecules that regulate synaptic formation, stabilization, and plasticity within these circuits.

In this framework, patchy/columnar projections correspond to spatially constrained “wiring” architectures, whereas diffuse projections provide a substrate for distributed modulation and integration.

### 5.2 Molecular candidates and hierarchical organization

Axon guidance and targeting systems (e.g., ephrin/Eph signaling, semaphorin pathways) are strong candidates for “wiring” mechanisms, consistent with their roles in topographic mapping and projection specificity (Drescher et al., 1995; Cheng et al., 1995; Cang et al., 2005; Triplett and Feldheim, 2011).

Synaptic organizers (e.g., CBLN2 and associated NRXN-GRID complexes) regulate dendritic spine formation and synaptic connectivity, consistent with “plug-in” functions (Shibata et al., 2021b; Sanes and Zipursky, 2020).

### 5.3 Integration across scales

Here, “intrinsic elaboration” in PFC does not imply independence from neural activity. Rather, it refers to a developmental condition in which intrinsic molecular programs contribute more strongly to cortical organization under relatively weak external sensory constraints (e.g., Ataman et al., 2016). Activity-dependent modulators identified in the primate visual cortex, including OCC1-related systems (Takahata et al., 2009), may also contribute to stabilization of cortical circuitry. All cortices use both activity-dependent and molecular mechanisms with different relative contributions. In primary sensory systems, topographic relationships are formed between peripheral inputs and cortical targets via ephrins/Eph (Dresher et al., 1995; Cheng et al., 1995). The precision of these relationships is closely linked to spontaneous retinal waves in the developing visual system (McLaughlin et al., 2003; Huberman et al., 2008). Activity-dependent mechanisms also regulate odorant receptor-specific axon sorting in the olfactory system (Serizawa et al., 2006). In contrast, the primate PFC operates within a multimodal and highly recurrent association network in which inputs and projection targets do not exhibit simple point-to-point topographic correspondence. We therefore propose that activity-dependent refinement in the PFC may operate under different organizational constraints, interacting with intrinsic molecular programs to stabilize large-scale association architectures rather than precise sensory maps.

Importantly, the correspondence between projection architecture and molecular systems is inferred from multiple convergent datasets. The present model is therefore based on the integration of:

(1) Mesoscale anatomical connectivity (Watakabe et al., 2023),
(2) spatial transcriptomic gradients reflecting cell-type organization across cortical regions (Chen et al., 2023),
(3) developmental molecular mechanisms (Zhang et al., 2026), and
(4) single-neuron structural analyses demonstrating refined projection specificity (Gou et al., 2025).

Together, these datasets support a multi-scale framework linking:

molecular signaling → dendritic and synaptic structure → columnar organization → large-scale connectivity.

### 5.4 Limitations and testable predictions

We emphasize that the molecular assignments proposed here are hypothesis-driven and not direct experimental demonstrations. In particular, the causal relationship between specific molecular pathways and columnar connectivity patterns remains unresolved.

This framework generates several testable predictions:

Manipulation of candidate “wiring” molecules should alter spatial organization of projection patterns,

Modulation of “plug-in” molecules should affect spine density and synaptic integration without altering gross projection topology,

Spatial transcriptomic gradients should predict local connectivity motifs at the columnar scale.

Future studies combining tracer mapping, genetic manipulation, and spatial omics will be required to test these predictions (Sanes and Zipursky, 2020). Importantly, in this context, the present model is best understood as a hypothesis-generating framework rather than a definitive mechanistic conclusion.

### 5.5 Implications

This dual-control framework can be contrasted with established developmental logics in other brain systems (Cang et al., 2005; Mori and Sakano, 2011; Förster et al., 2006; see Supplementary Table 3), and thus provides a conceptual bridge between classical columnar theories and modern molecular neuroscience. By linking structural connectivity with gene regulatory programs, it offers a unified perspective on how primate-specific cortical organization may contribute to cognitive specialization. Taken together, these observations suggest a potential multi-scale correspondence between molecular signaling and cortical connectivity.

## 6 Discussion

The present review integrates classical anatomical studies with recent molecular and transcriptomic advances to provide a multi-scale perspective on the columnar organization of the primate prefrontal cortex (PFC). Across these levels, a consistent theme emerges: cortical organization is not defined by a single structural unit, but by a hierarchical system spanning dendritic architecture, cellular complexity, and large-scale connectivity.

As outlined in Section 1, at the microanatomical level, vertically organized dendritic structures and microcolumns provide a repeating structural framework. Quantitative studies have demonstrated that these units vary across cortical regions, particularly in dendritic complexity and spine density, which are markedly enhanced in association and prefrontal cortices. At the systems level, tracer studies have revealed that cortical connectivity is organized into both spatially restricted (patchy) and distributed (diffuse) projection patterns, suggesting a multi-scale architecture linking local modules with long-range networks (Watakabe et al., 2023).

At the cellular level, the spine-density hierarchy described by Elston et al. indicates that pyramidal neurons in higher-order cortices possess greater dendritic arborization and synaptic capacity (see Section 2). This gradient is complemented by developmental findings showing prolonged synaptogenesis and delayed pruning in the human PFC, reflecting extended plasticity and functional refinement. Recent transcriptomic studies further demonstrate that these structural and developmental features are supported by coordinated gene expression programs, including those regulating synapse formation, neuronal differentiation, and glial interactions (see Section 3).

Despite these advances, several fundamental questions remain unresolved. First, the relationship between microcolumnar structure and functional computation is still debated, and it is unclear whether microcolumns represent discrete functional units or emergent properties of overlapping dendritic fields. Second, the correspondence between anatomical connectivity patterns and molecular signaling pathways is largely inferred rather than directly demonstrated. Third, the causal mechanisms linking gene expression gradients to columnar architecture and cognitive function remain to be established.

To address these issues, we proposed a dual-control model in which cortical organization is governed by two interacting systems (see Section 4): (i) spatially structured “wiring” mechanisms that establish projection patterns, and (ii) “plug-in” mechanisms that regulate synaptic formation and plasticity. Importantly, this model is not presented as a definitive mechanistic explanation, but as a hypothesis-generating framework derived from the integration of anatomical, molecular, and developmental data. Its primary value lies in generating testable predictions that can be evaluated experimentally. A conceptual comparison of these organizational principles across major brain systems is provided in Supplementary Table 3.

These observations are consistent with recent single-neuron projectome studies showing refined and selective axon targeting in the primate PFC (Gou et al., 2025), supporting the notion of highly structured connectivity at the microscale.

Future research should focus on directly linking these levels of organization. Combining high-resolution connectomics, spatial transcriptomics, and targeted molecular manipulation will be essential to determine whether specific molecular pathways causally regulate columnar architecture. In particular, experimental perturbation of candidate guidance molecules and synaptic regulators may clarify their roles in shaping cortical organization. In addition, cross-species comparative studies will be critical for identifying primate-specific mechanisms underlying prefrontal expansion and cognitive specialization.

In summary, the primate PFC exhibits a hierarchical organization that emerges from the interaction of structural, cellular, and molecular processes. Understanding how these processes are coordinated across scales will be essential for elucidating the neural basis of higher cognitive function and its evolutionary origins. The primary value of the present framework lies in generating experimentally testable predictions linking molecular signaling, synaptic organization, and large-scale cortical connectivity.
