# Supplementary material for: Intrinsic elaboration of prefrontal modularity: a dual-control model of axon bundling and synaptic docking
Source: Front Neuroanat. 2026 Jun 2;20:1761080. doi: 10.3389/fnana.2026.1761080 (PMC13269059; doi:10.3389/fnana.2026.1761080)
Supplement: Supplementary file 1 [file Table_1.DOCX]

| Supplementary Table 1 | | | | | |
| --- | --- | --- | --- | --- | --- |
| Molecular system | Representative molecules | Proposed contributions | Supporting evidence | Evidence level | Key references |
| Axon guidance systems | ROBO2, ephrins/Ephs, semaphorins | Fasciculation, projection targeting, topographic organization | Known developmental guidance roles, spatial enrichment patterns | Inferred | Drescher et al., 1995; Cheng et al.,1995;  Cang et al., 2005; Triplett & Feldheim, 2011 |
| Cell-adhesion systems | PCDH11X, PCDH17, cadherins (CDH8 etc.,) | Stabilization of clustered projections and local modularity | Spatial transcriptomic enrichment; adhesion functions | Inferred | Chen et al., 2023 |
| Synaptic organizers | CBLN2, NRXN-GRID complexes | Synapse formation and spine stabilization | Functional experimental evidence | Supported | Shibata et al., 2021b |
| Synaptic maturation systems | SYT10 and related synaptic genes | Refinement and maturation of local circuitry | Develop-mental transcript-mics | Inferred | Zhang et al., 2026 |
| Activity-related modulators | OCC1/FSTL1, testican-related genes | Activity-dependent stabilization, ECM modulation and dendritic arborization | Visual cortical gradient studies or prefrontal specific | Supported/inferred | Takahata et al., 2009; Ataman et al., 2016 |

**Supplementary Table 1. Candidate molecular systems potentially related to intrinsic refinement and modular organization in the primate PFC.**

Summarizes candidate molecular systems discussed in relation to intrinsic refinement and modular organization in the primate PFC. Candidate assignments are derived from convergent evidence including developmental studies, spatial transcriptomics, connectomics, and known molecular functions in axon guidance, cell adhesion, or synaptic organization. Inclusion of a molecule or molecular family does not imply direct experimental demonstration of its causal role in prefrontal column formation.
