# Supplementary material for: Intrinsic elaboration of prefrontal modularity: a dual-control model of axon bundling and synaptic docking
Source: Front Neuroanat. 2026 Jun 2;20:1761080. doi: 10.3389/fnana.2026.1761080 (PMC13269059; doi:10.3389/fnana.2026.1761080)
Supplement: Supplementary file 2 [file Table_2.DOCX]

| **Supplementary Table 2** | | | | | |
| --- | --- | --- | --- | --- | --- |
| **Dataset/**  **study** | **Species** | **Methods** | **Main finding** | **Relevance to present model** | **Evidence Model** |
| Watakabe et al., 2023 | marmoset | tracer mapping | Patchy and diffuse projection systems | Connectivity architecture | Supported |
| Chen et al., 2023 | macaque | spatial transcript-mics | Region- and layer-specific gene organization | Molecular gradients | Supported |
| Zhang et al., 2026 | human/  macaque | Spatio-temporal transcript-mics | Developmental stripe-like expression domains | Develop-mental refinement | Supported |
| Shibata et al., 2021a | mouse/  primate comparison | Develop-mental genetics | RA signaling regulates PFC patterning | Molecular signaling | Supported |
| Shibata et al., 2021b | primate/  human | enhancer + functional analysis | CBLN2-driven spinogenesis | Plug-in systems | Supported |
| Gou et al., 2025 | macaque | single-neuron projections | Refined axon targeting | Projection specificity | Supported |
| Yuan et al., 2024 | mouse | BARseq connect-mics | Spatially resolved single-neuron projections | Nested modularity framework | Conceptual support |

**Supplementary Table 2. Representative transcriptomic, connectomic, and developmental datasets relevant to cortical modularity and intrinsic refinement.**

Supplementary Table 2 summarizes representative datasets discussed in the present review. These studies provide convergent evidence relevant to cortical modularity, intrinsic refinement, and hierarchical organization in the primate PFC. The present review integrates these datasets conceptually and does not claim direct mechanistic linkage between all listed findings. The similarity of projections between the prefrontal cortex and multiple brain regions across primates has been confirmed through comparisons with studies such as Watakabe et al. (2023) and Schmahmann and Pandya (2006).
