# Supplementary material for: Intrinsic elaboration of prefrontal modularity: a dual-control model of axon bundling and synaptic docking
Source: Front Neuroanat. 2026 Jun 2;20:1761080. doi: 10.3389/fnana.2026.1761080 (PMC13269059; doi:10.3389/fnana.2026.1761080)
Supplement: Supplementary file 3 [file Table_3.DOCX]

| **Supplementary Table 3** | | | | |
| --- | --- | --- | --- | --- |
| **System** | **Principle determinants** | | **Primary Molecular Codes** | **Role of External Activity** |
| Visual | Gradient and activity-dependent tuning of synapse connectivity | ephrins/ Ephs, Draxin, | | Retinal waves are essential for projection refinement. |
| Olfactory | Point-driven (Receptor-sorting) | ORs, ephrin/EphA5, Kirrel2/3  PCDH8 | | Receptor-dependent alignment |
| Hippocampus | Layer-driven (Laminar) | Nectins, Cadherins, Reelin | | Structural organization |
| PFC (8Ad, 8Av, 8b) | Dual-Control (Intrinsic Elaboration) | PCDHs, CDHs, CBLN2 | | Stronger intrinsic molecular contribution with activity-dependent refinement |

**Supplementary Table 3. Comparison of Developmental Logics Across Brain Systems.**

This comparative matrix defines the unique features of the PFC's "Intrinsic Elaboration" logic in contrast to established neurodevelopmental models studied primarily in rodents. By juxtaposing the "map-driven" logic of the visual system, the "point-driven" logic of the olfactory system, and the "layer-driven" logic of the hippocampus, we characterize the PFC’s Dual-Control Model as a high-dimensional association framework. This table supports the argument in Section 4 that the primate PFC reconfigures a shared vertebrate genetic toolbox into a distinct association architecture under relatively weak external sensory constraints.
